# A longitudinal study on the effect of extreme temperature on non-accidental deaths in Hulunbuir City based on DLNM model

**DOI:** 10.1007/s00420-023-01986-5

**Published:** 2023-06-03

**Authors:** Sheng Gao, Tian Yang, Xiuhong Zhang, Guofeng Li, Yuhan Qin, Xiangnan Zhang, Jing Li, Shengmei Yang, Minghui Yin, Jufang Zhao, Nana Wei, Jing Zhao, Li li, Huan Li, Xuanzhi Yue, Wenyu Zhang, Xinrui Jia, Yaochun Fan, Hongli Liu

**Affiliations:** 1 Institute of Artificial Intelligence, School of Electrical and Information Engineering, Hunan University, Changsha, 410000 People’s Republic of China; 2 Inner Mongolia Autonomous Region Center for Disease Control and Prevention, Hohhot, 010070 People’s Republic of China; 3 Inner Mongolia Medical University, Hohhot, 010107 People’s Republic of China

**Keywords:** Extreme temperature, Death, Distributed lag nonlinear model, Time series

## Abstract

**Objective:**

To explore the frequency and effect of extreme temperature on the non-accidental death rate in Hulunbuir, a Chinese ice city.

**Methods:**

From 2014 to 2018, mortality data of residents residing in Hulunbuir City were collected. The lag and cumulative effects of extreme temperature conditions on non-accidental death and respiratory and circulatory diseases were analyzed by distributed lag non-linear models (DLNM).

**Results:**

The risk of death was the highest during high-temperature conditions, the RR value was 1.111 (95% CI 1.031 ~ 1.198). The effect was severe and acute. The risk of death during extreme low-temperature conditions peaked on the fifth day, (RR 1.057; 95% CI 1.012 ~ 1.112), then decreased and was maintained for 12 days. The cumulative RR value was 1.289 (95% CI 1.045 ~ 1.589). Heat significantly influenced the incidence of non-accidental death in both men (RR 1.187; 95% CI 1.059–1.331) and women (RR 1.252; 95% CI 1.085–1.445).

**Conclusions:**

Regardless of the temperature effect, the risk of death in the elderly group (≥ 65 years) was significantly higher than that of the young group (0–64 years). High-temperature and low-temperature conditions can contribute to the increased number of deaths in Hulunbei. While high-temperature has an acute effect, low-temperature has a lagging effect. Elderly and women, as well as people with circulatory diseases, are more sensitive to extreme temperatures.

## Introduction

With the profound changes in the global climate, extreme high- and low-temperature conditions are becoming more and more frequent around the world. Many epidemiological studies have shown that extremes in temperature can increase the risk of mortality (Hu et al. 2008; Intergovernmental Panel on Climate Change 2013; Kan et al. 2007); however, the data and conclusions drawn from different regions are often different. Hulunbuir is a Chinese city located in Inner Mongolia and is characterized by a temperate continental climate, with long cold winters and short hot summers. Additionally, it is known as one of the few “ice and snow cities” of China. In this study, longitudinal meteorological data and daily population mortality in Hulunbuir City from 2014 to 2018 were analyzed to quantitatively evaluate the effects of extreme temperatures, both high and low, on un-accidental death. Using these data, this study aims to provide a scientific basis for the prevention and control of meteorological sensitive diseases in the regional population.

## Materials and methods

### Data sources

From 2014 to 2018, mortality data of Hulunbuir City residents were collected from the Center for Disease Control and Prevention of Hulunbuir City. These data included gender, age, date of death, the root cause of death, and the International Classification of Diseases (ICD-10) code that was associated per case. According to the ICD-10 code, “total deaths” in this study refer to non-accidental deaths that exclude injury, poisoning, and other external causes. The code of non-accidental death is A00 ~ R99, the code of circulatory disease death is I00 ~ I99, and the code of respiratory disease death is J00 ~ J99. To identify susceptible populations, total mortality was stratified according to sex (male/female) and age (0–64 years and ≥ 65 years).

All meteorological data were collected from the China Meteorological Science data sharing Service Network and included daily average temperature, mean air pressure, relative humidity, and wind speed. The air quality data in the same period was collected from the environmental monitoring station of Hulunbuir City and included daily PM_10_, SO_2_, and NO_2_ levels.

### Statistical analysis

#### Modeling

As the daily death toll of residents obeys a Poisson distribution and the relationship between temperature and death is usually non-linear, this study used a Poisson generalized additive model (GAM) (Peng et al. 2006) and distributed lag non-linear models (DLNM) (Gasparrini 2014) to analyze the effect of extreme temperature on residents’ death. Using these models, confounding factors, such as air pollutants, long-term trends, and the day of the week effect, were controlled. The basic model of DLNM is as follows:

$${\text{Log}}\left[ {E\left( {{\text{Yt}}} \right)} \right]\, = \,\alpha \, + \,cb\left( {{\text{Tt}},{\text{ lag}}\, = \,{\text{maxlag}}} \right)\, + \,{\text{NS}}\left( {{\text{AIR}},{\text{ df}}} \right)\, + \,{\text{NS}}\left( {{\text{Time}},{\text{ df}}} \right)\, + \,{\text{DOWt}}.$$

In the formula, *E* (Yt) is the number of daily deaths of *t* days; *α* is intercept; *β* is the coefficient of exposure response, that is, the increase of daily mortality caused by each unit of temperature change; Tt is the cross basis matrix of daily average temperature and lag days; lag is lag days. NS (AIR,df) is the cubic spline function of air pollutants, and the degree of freedom of df is 3th time is the long-term trend of time. Choosing the appropriate DF value for the date can effectively control the long-term fluctuation and seasonal fluctuation trend of meteorological factors-death series data; in this study, df is 7/year, and DOW represents the day of the week effect.

**Fig. 1:**
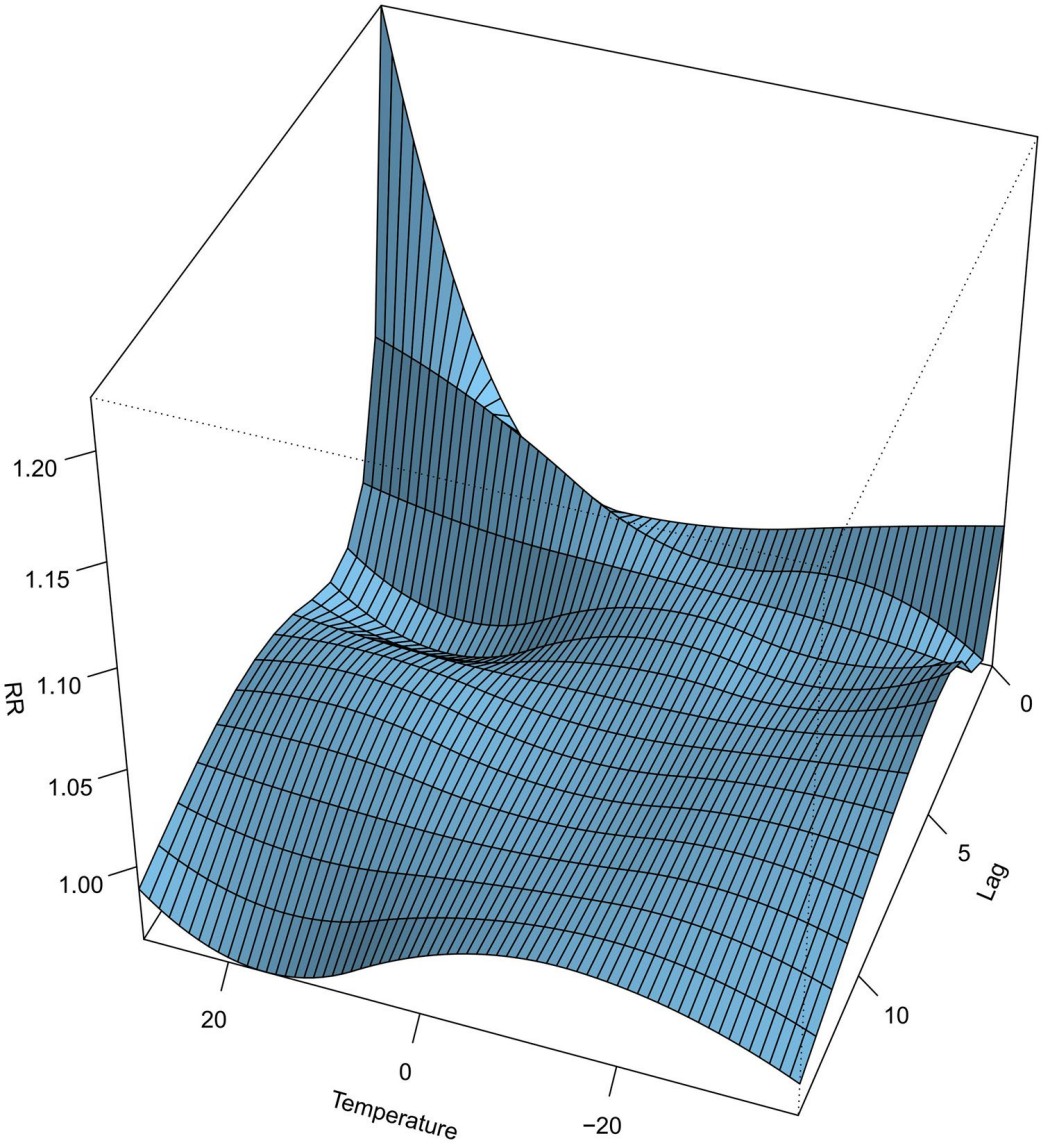
Three-dimensional diagram of different lag days of the average temperature on residents’ death

According to the calculation, the Pearson correlation coefficient between PM_10_, SO_2_, and NO_2_ was 0.352–0.504 which indicated that the collinearity of pollutants in this study is negligible (Gasparrini 2011). That is, after controlling different pollutants, the results were similar (Fig. 1; Table 1). Previous studies (Wong et al. 2008; Samoli et al. 2008) have found that air pollutants had the closest relationship to death on the same day and the previous day, so the moving mean value (Lag01) of the PM_10_ concentration on the same day and the previous day was included in the model.

**Table 1:** General situation of daily deaths, meteorological factors, and air pollutants of residents in Hulunbuir City from 2014 to 2018

Variable	$\overline{x} \pm s$	Minimum	*P*_25_	*P*_50_	*P*_*7*5_	Maximum
Total deaths/daily	47.86 ± 8.39	25	42	48	53	79
*Disease type*						
Daily death toll of respiratory diseases/person	3.00 ± 1.83	0	2	3	4	11
Daily death toll of circulatory diseases/person	26.31 ± 6.13	9	33	26	30	52
*Gender*						
Male	29.29 ± 6.16	12	25	29	33	55
Female	18.57 ± 4.84	4	15	18	22	35
*Age*						
0–64 years	18.74 ± 4.75	6	16	19	22	36
≥ 65 years old	29.12 ± 6.41	10	25	29	33	54
*Meteorological factors*						
Mean air temperature (°C)	− 0.17 ± 17.35	− 38.8	− 17.5	2.8	15.8	29.5
Mean air pressure (hpa)	939.77 ± 7.69	918.4	934.1	939.4	945.5	962.5
Relative humidity (%)	61.94 ± 15.12	15	52	66	73	94
Wind speed (m/s)	4.40 ± 1.91	1.1	3.0	4.1	5.5	15.4
*Air pollutants*						
SO_2_ (μg/m^3^)	7.21 ± 6.18	1.0	3	5	10	58.0
NO_2_ (μg/m^3^)	18.42 ± 9.75	1.5	11	16	23	69.0
PM_10_ (μg/m^3^)	49.27 ± 34.69	10.0	28	42	60	676.0

15 °C was used as the optimum ambient temperature (MMT) during the study period. At this temperature, the lowest mortality was observed. Therefore, an MMT of 15 °C was used as the reference temperature for all subsequent modeling. To estimate the cold effect of air temperature, the first percentile of the daily average temperature (− 33 °C) was defined as the baseline for extreme low-temperature conditions. Conversely, to estimate the thermal effect of air temperature, the 99th percentile of daily average temperature (27 °C) was defined as the baseline for extreme high-temperature conditions. The maximum lag days were set to 14 days.

#### Statistical software

R (4.0.0) software is used for statistical analysis, and the nonlinear model of distributed lag is established using the “splines (Gasparrini 2014)” and “dlnm (Gasparrini 2014)” toolkits. *P* values < 0.05 were statistically significant.

## Results and discussion

### General situation

From 2014 to 2018, 87,393 non-accidental deaths were recorded among residents in Hulunbuir City. The average daily death toll was 47.86 ± 8.39. Of the total mortality, approximately 48,037 (54.97%) and 5479 (6.27%) deaths were associated with circulatory and respiratory diseases, respectively. Further, 38.81% were females and 61.19% were males. Additionally, 39.16% of all deaths were recorded in individuals aged 0–64 years and 60.84% were in persons > 65 years. During the same period, the daily average temperature, average air pressure, relative humidity, and wind speed were − 0.17 °C, 939.77 hpa, 61.94% and, 4.40 m/s, respectively. The average daily concentrations of SO_2_, NO_2_, and PM_10_ were 7.21 μg/m^3^, 18.42 μg/m^3^, and 49.27 μg/m^3^, respectively (Table 1).

### Relationship between daily average temperature and none-accidental death

**Fig. 2:**
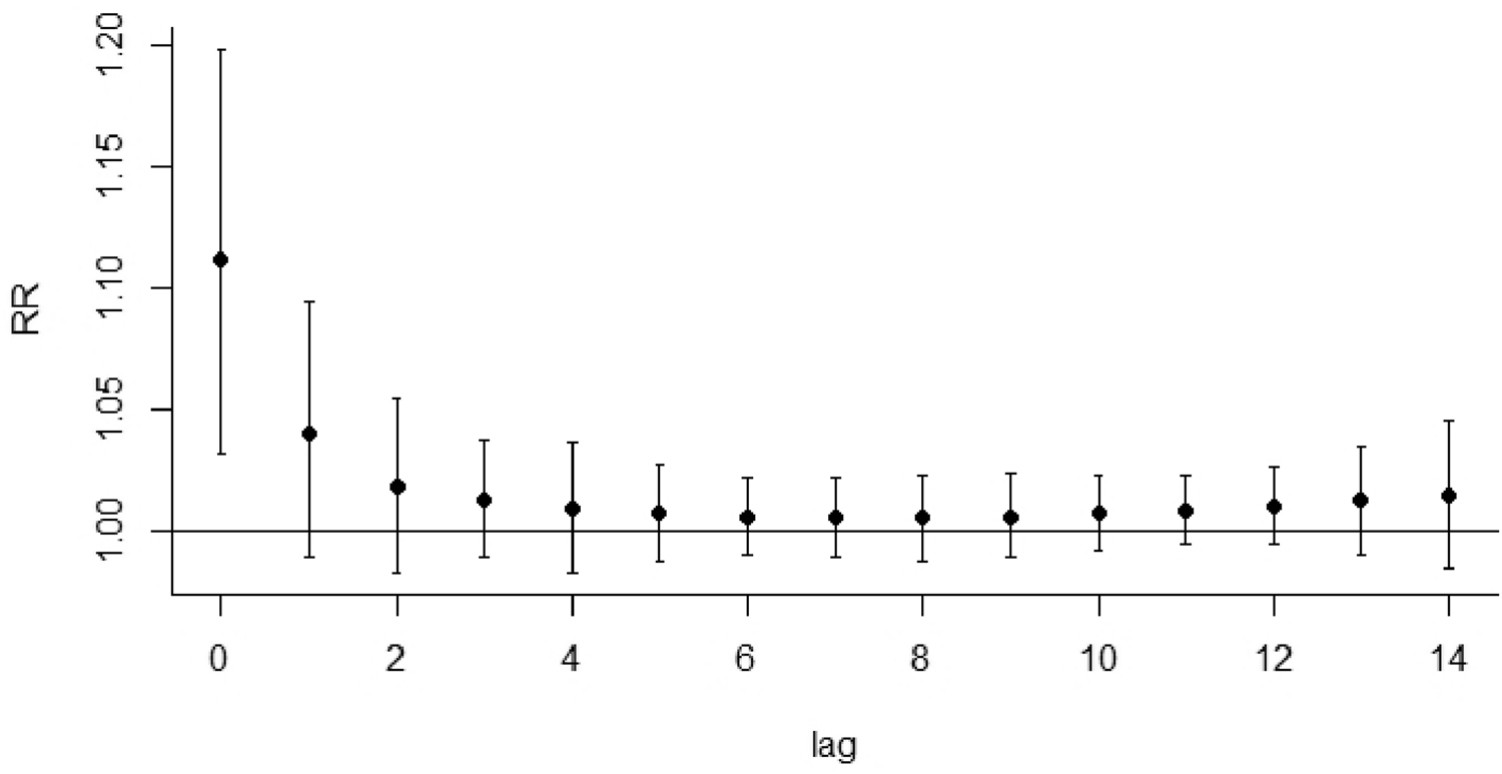
Lag effect of extreme high-temperature conditions on total non-accidental deaths (reference temperature: 15 °C)

**Fig. 3:**
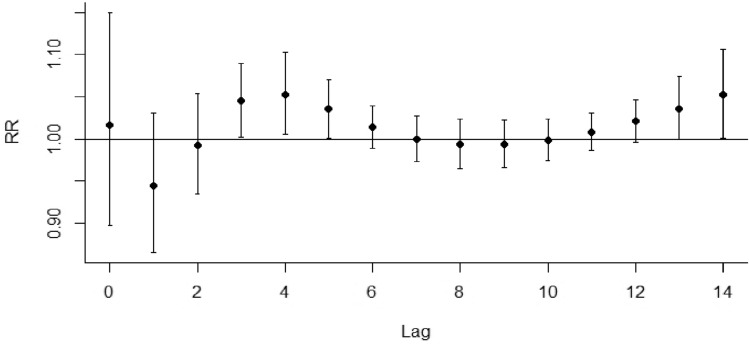
Lag effect of extreme low-temperature conditions on total non-accidental deaths (reference temperature: 15 °C)

Initially, the relationship between daily average temperature, lag time, and daily the un-accidental death rate of residents was assessed. The data from these analyses indicate that the relationship between non-accidental death and temperature is nonlinear and that both extremely high temperatures and low-temperature conditions are associated with an increased risk of death (Fig. 1). Further, the effect of extremely high-temperature conditions on all daily causes of death is strong and short-lived. For instance, during high-temperature conditions, the risk of death is the highest with a risk ratio (RR) value of 1.111 (95% CI 1.031 ~ 1.198). For example, when the average daily temperature reaches 27 °C, the death rate of the whole population increases by 11.1% compared to the reference temperature (15 °C). The effect of high temperature usually lasts for approximately 5 days with a cumulative RR of 1.212 (95% CI 1.104 ~ 1.331) (Fig. 2, Table 2). In contrast, the effect of low temperature on population death was slow and sustained. An increase in non-accidental deaths was observable on the second day following the incidence of low temperatures and lasted for approximately 12 days. The risk of death peaked on the fifth day with a maximum RR of 1.057 (95% CI 1.012 ~ 1.112) and a cumulative RR of 1.289 (95% CI 1.045 ~ 1.589) (Fig. 3, Table2).

**Table 2:** The cumulative lag effect of extreme temperature conditions on population death in Hulunbuir City from 2014 to 2018

Variable	Thermal effect (lag0–5) RR (95% CI)	Cold effect (lag2–14) RR (95% CI)
The whole population	1.212 (1.104 ~ 1.331)	1.289 (1.045 ~ 1.589)
*Disease type*		
Respiratory diseases	1.214 (0.865 ~ 1.705)	0.993 (0.485 ~ 2.037)
Circulatory diseases	1.267 (1.120 ~ 1.434)	1.531 (1.168 ~ 2.007)
*Gender*		
Male	1.187 (1.059 ~ 1.331)	1.249 (0.996 ~ 1.616)
Female	1.252 (1.085 ~ 1.445)	1.350 (0.982 ~ 1.856)
*Age*		
0–64 years	1.209 (1.053 ~ 1.388)	1.022 (0.745 ~ 1.402)
≥ 65 years	1.215 (1.080 ~ 1.399)	1.487 (1.149 ~ 1.925)

### Effect of temperature on non-accidental death of residents of different diseases and age groups

Extreme temperatures had no significant effect on respiratory disease-associated mortality; however, both extremely high temperatures and low-temperature conditions were significantly associated with an increased incidence of deaths due to circulatory diseases. The cumulative RR values of high-temperature and low-temperature conditions were 1.267 (1.120–1.434) and 1.531 (1.168–2.007), respectively. Heat significantly influenced the incidence of deaths in both men and women, with higher death rates being observed in women relative to men. Regardless of temperature, the risk of death in the elderly group (≥ 65 years) was significantly higher than that in the young group (0–64 years) (*P* < 0.05) (Table 2).

## Discussion

In this study, the longitudinal effect of extreme high and low temperatures on the incidence of non-accidental death in Hulunbuir City was assessed using distributed lag nonlinear modeling. The maximum lag days were set to 14 days, and the model showed that the optimum ambient temperature was 15 °C, which was close to the 75% quantile of the daily average temperature during the study period. Furthermore, this result is consistent with data reported by Ma Wenjun's (Ma et al. 2015) study of 66 communities in China and Curriero's (Curriero et al. 2002) study based on 11 cities in the eastern United States.

Here, we show that in Hulunbuir City, both extremely high-temperature and extreme low-temperature conditions will increase the total number of deaths per day; however, the risk of death is the highest on days of high-temperature weather. During such periods, the effect of high temperature on death is short-lived but severe (acute effect). In contrast, low-temperature conditions have a lag effect on the incidence of non-accidental deaths. Our data indicate that the risk of death increases gradually with an increase of lag days, reaching the maximum on the 4th and 5th days. Similar to the research results of Hefe, (Tang et al. 2018) this effect was observed to last for approximately 12 days. In agreement with this finding, Ma Xinming (Ma et al. 2016) and others found that the lag effect of extreme low-temperature conditions on the incidence of non-accidental deaths reached the maximum at approximately 5–10 days. However, the authors noted that different cities had different lag effects and suggested that regional differences should be taken into account when formulating population health prevention and control measures to extreme weather conditions.

This study found that both extreme high-temperature and extreme low-temperature conditions are associated with an increased incidence of non-accidental deaths due to circulatory diseases. Furthermore, this effect was more pronounced in low-temperature conditions relative to high-temperature conditions. Li Yonghong (Li et al. 2018) and others analyzed the effect of extreme temperature on cardiovascular and cerebrovascular death in Chongqing. During extreme high-temperature conditions, those authors found that when the temperature increases by 1 °C, the mortality rate from cardiovascular disease increased by 2.2%. At low-temperature conditions, when the temperature decreased by 1 °C, the cardiovascular disease mortality rate decreased by 6.1%. Interestingly, several international studies have shown that extreme high-temperature or low-temperature conditions can affect the levels of dopamine, epinephrine, and other related factors in the body as well as increase the permeability of the blood–brain barrier (Ruan et al. 2013; Zheng et al. 2016). These effects should be further investigated in future studies.

This study aimed to identify population sensitive to extreme temperature conditions. Our survey data show that people aged over 65 accounted for approximately 60.84% of the total deaths in Hulunbei. Additionally, our data indicate that extreme temperature, both hot and cold, significantly influenced the death rate of elderly individuals (> 65 years) relative to younger individuals (0–64 years). These data show that extreme temperature conditions may contribute to a large number of deaths observed in the elderly population; however, this may need to be fully verified in future work. When stratified for gender, extreme high-temperature conditions are associated with an increased mortality rate in women relative to men. These findings are consistent with both Chinese and other international studies (Basu and Ostro 2008). Together, these data indicate that both the elderly and women are more sensitive to high-temperature weather conditions. Furthermore, these data highlight the importance of preventative strategies to mitigate non-accidental death, such as avoiding going out for prolonged periods and outdoor activities during extreme temperature conditions. These recommendations aim to improve public health services and strengthen the construction of infrastructure and equipment to effectively improve the ability of sensitive populations to cope with extreme weather and reduce associated mortality.

## Conclusion

This study has several limitations. First, our analysis uses the daily average temperature which may introduce ecological bias. Second, this study only analyzed the data of one city. For more robust analysis and conclusions, several cities should be assessed to be more representative of the different regions and cities in China.

## Data Availability

Data were collected from administrative departments rather than human subjects. No personal private information was involved.
